# Multiple sclerosis and the risk of infection: considerations in the threat of the novel coronavirus, COVID-19/SARS-CoV-2

**DOI:** 10.1007/s00415-020-09822-3

**Published:** 2020-04-17

**Authors:** M. D. Willis, N. P. Robertson

**Affiliations:** 1 Department of Neurology, University Hospital Wales, Cardiff, CF14 4XN UK; 2 Institute of Psychological Medicine and Clinical Neuroscience, Cardiff University, University Hospital of Wales, Heath Park, Cardiff, CF14 4XN UK

## Introduction

Global healthcare systems are rapidly having to change and adapt in the face of the COVID-19 pandemic. At a more individual level, neurologists are being faced with complex decisions regarding the risk of infection in specific patient groups, and in particular those receiving immunosuppressant or immunomodulatory therapy. One such important patient group is those individuals with multiple sclerosis (MS) receiving disease-modifying therapies (DMTs).

It has previously been established that people with MS (PwMS) have an increased risk of infections compared with the general population. These infections can lead to significant morbidity and may also contribute to relapses and a worsening of neurological symptoms. First-generation DMTs such as interferon-beta (IFN-β) or glatiramer acetate (GA) are not thought to be associated with a significantly increased risk of infection, although more efficacious second generation DMTs have demonstrated a higher risk profile. However, the majority of these observations are derived from clinical trials of MS DMTs which, because of restricted inclusion criteria and short-term follow-up, may underestimate the overall risk. Real-world experience with longer term follow-up in representative patient groups may be necessary to achieve a more accurate picture, although these data are currently limited. In the context of the evolving COVID-19 pandemic, it has become particularly important to understand these risks to make informed decisions regarding treatment and to be able to communicate this information effectively to patients. In all cases any increased risk of infection and associated morbidity will need to be carefully balanced against risks of stopping treatment and rebound disease activity.

From an historical perspective, it is also interesting to note that the coronavirus family has been previously investigated for a potential association with MS, and more recently has been utilised to make a mouse model of the disease (coronavirus-induced encephalomyelitis). We should also not assume that effects of COVID-19 infection, and in particular the rarer but more serious secondary hyperinflammation syndrome, should necessarily be worsened in PwMS on DMTs and they may even be protective; inhaled treatment with interferon-beta as well as Fingolimod are currently under investigation as a potential treatment for COVID-19 infection. Nevertheless, understanding the risk of infection in MS patients remains important, especially with regard to decisions concerning therapeutic management.

In this month’s journal club, we consider three papers relevant to the risk of infection in patients with MS. These studies highlight the general increased risk of infections in PwMS and those of specific treatments. Although data from many DMTs are considered in these studies, there are some—notably alemtuzumab and ocrelizumab that are not currently captured by these predominantly registry-based studies. In the meantime, many national neurology associations as well as MS charitable bodies have issued guidance with regard to the use of all DMTs in light of the current pandemic and are a useful practical resource for neurologists to review.

## Infections in patients diagnosed with multiple sclerosis: a multi-database study

This study aimed to characterise the infection risk of patients with MS compared with a cohort of patients without MS. The authors utilised two large databases, the United States Department of Defense (US-DOD) health care system and the United Kingdom's Clinical Practice Research Datalink GOLD (UK-CPRD). The US-DOD is composed of data from members of the US-DOD, retirees and dependents. The UK-CPRD is a large, prospectively collected medical record database from over 500 general practices.

Patients with a first diagnosis of MS were identified between the years 2001 and 2016 (UK-CPRD, *n* = 6932) and 2004 and 2017 (US-DOD, *n* = 8695). MS patients were matched to patients without MS based on age, sex, date of MS diagnosis/matched date, and location (US-DOD; 86,934, UK-CPRD 68,526). Estimated incidence rates (IRs) and incidence ratios (IRRs) with 95% confidence intervals of the first of each infection outcome were calculated. MS patients in the US-DOD had received at least one MS DMT, but information on treatment in the UK-CPRD was lacking.

The incidence of infection was higher in MS patients compared with non-MS patients: US-DOD (IRR 1.76; 95% CI 1.72–1.80) and UK-CPRD (IRR 1.25; 95% CI 1.21–1.29). Compared to patients without MS, the rate of infections causing hospitalisation in MS patients was higher in both databases (US-DOD IRR 2.43; 95% CI 2.23–2.63 and UK-CPRD IRR 2.00; 95% CI 1.84–2.17). With regard to rates of infection by site, MS patients had higher rates of urinary and kidney infections compared with non-MS patients (US-DOD IRR 1.88; 95% CI 1.81–1.95 and UK-CPRD IRR 1.97; 95% CI 1.86–2.09), with female MS patients having a higher risk. Pneumonia and influenza risk were increased in MS patients in the US-DOD, but not the UK-CPRD while skin and fungal infections were elevated across both databases. Viral infections were slightly increased in the US-DOD cohort, but not the UK-CPRD. Respiratory and throat infections rate were slightly elevated in the MS group. There was a slightly elevated rate of any opportunistic infections (US-DOD IRR 1.54; 95% CI 1.48–1.61 and UK-CPRD IRR 1.35; 95% CI 1.26–1.45), the majority of which were candidiasis and herpes viruses. IRs of meningitis and encephalitis, tuberculosis, hepatitis B and hepatitis C were low across both databases. Patients using monoclonal antibodies (identified in the US-DOD) had a lower rate of infection compared to all MS patients but the rate of hospitalised infections was higher.

*Comment *This study utilised large databases with a long follow-up time. MS patients were demonstrated to be at an increased risk generally of infections, and of infections requiring hospitalisation. It is pertinent to observe that because of lack of available data, this study was unable to focus on the risk of infection with individual DMTs or in MS patients not on treatment. Other limitations due to lack of data included the inability to account for relevant covariates and being unable to determine if some infections required hospitalisation.

Persson et al. Mult Scler Relat Disord. 2020;41:101–982. 10.1016/j.msard.2020.101982. [Epub ahead of print].

## Infection risks among patients with multiple sclerosis treated with fingolimod, natalizumab, rituximab, and injectable therapies

This registry study aimed to examine the risk of serious infections associated with routinely used MS DMTs as well as rituximab, which is commonly used in this population. The Swedish MS Register was utilised to identify all patients with relapsing–remitting MS and who started a treatment with IFN-β and GA, fingolimod, natalizumab, or rituximab between 2011 and 2017. Five comparator patients were matched for each MS patient from the general population by age, sex and region. The main outcome was identified as the time until the first serious infection (hospitalisation caused by the infection). Less severe infections were identified by prescriptions of any systemic antibiotic and antiviral medication for herpetic infections. Cox proportional hazard models were used to estimate hazard ratios. As participants could contribute data to multiple treatment cohorts, robust 95% confidence intervals were calculated.

A total of 8600 treatment episodes were included from a total of 6421 patients (female 71.9%; 2217 initiations of IFN-β and GA, 1535 fingolimod, 1588 natalizumab, and 3260 rituximab). The mean time (years) receiving DMTs was as follows: IFN-β and GA, 2.1; fingolimod, 7; natalizumab, 2.5; rituximab, 2.0. The crude serious infection incidence rate was higher across all MS patients compared to the general population. Before adjusting for differences in patient characteristics, the incidence rate of serious infections was similar for patients treated with IFN-β and GA (incidence rate, 8.9 [95% CI 6.4–12.1] per 1000 person-years), and natalizumab (11.4 [95% CI 8.3–15.3] per 1000 person-years). This was lower than the rate for fingolimod (14.3 [95% CI 10.8–18.5] per 1000 person-years) and rituximab-treated patients (19.7 [95% CI 16.4–23.5] per 1000 person-years). In the most adjusted model taking into account potential confounders and when comparing against IFN-β and GA, only rituximab had a statistically significant increased risk (HR, 1.70 [95% CI 1.11–2.61]) although point estimates for fingolimod and natalizumab were still greater than 1.00 (HRs, 1.30 [95% CI 0.84–2.03] and 1.12 [95% CI 0.71–1.77], respectively). Rituximab and natalizumab had the highest rate of antibiotic use. The use of antiviral drugs was significantly higher in patients taking natalizumab and fingolimod when compared with either IFN-β and GA or rituximab. After adjustment, the rate of herpetic infections was similar when comparing rituximab with IFN-β and GA.

*Comment* This large cohort study provides further support that patients with MS being treated with DMTs are at a generally increased risk of infection, with rituximab associated with the highest rate of serious infections. The authors note that because of the non-availability of primary care data in the national registries, most minor infections were not included in the study. Data were also lacking on several potential confounders including body mass index, smoking status, and varicella vaccination status.

Luna et al. JAMA Neurol. 2019 Oct 7. 10.1001/jamaneurol.2019.3365. [Epub ahead of print].

## Disease-modifying drugs for multiple sclerosis and infection risk: a cohort study

This paper investigated the association between MS DMTs and risk of infections in a population-based retrospective cohort study. The primary study outcome was the hazard of infections, based on physician claims with secondary outcomes related to the risk of infection-related hospital admissions and individual infections. A proportional means model for recurrent events was used to examine the association between DMT exposure and infections. All analyses were adjusted for sex, age, socio-economic status and co-morbidities. DMTs were grouped into first generation (IFN-β or GA) or second generation (natalizumab, fingolimod, or dimethyl fumarate). Of note, no patients were taking teriflunomide, alemtuzumab or the generic beta-interferon-1b (Extavia).

The authors identified 6793 MS patients (73.6% female). Mean age at index demyelinating event was 45.4 years and mean follow-up 8.5 years. During the study period, 1716 patients (25.3%) were prescribed at least one DMT. Patients prescribed ≥ 2 different DMT groups totalled 458 (6.7%). The patients in each group were as follows: IFN-β (1386, 20.4%), GA (656, 9.7%), natalizumab (100, 1.5%), oral DMTs (98, 1.4%)—fingolimod (61, 0.9%), dimethyl fumarate (40, 0.6%). Exposure to any DMT, first generation DMT (either grouped or as IFN-β or GA separately) was not associated with an altered hazard (aHR) for an infection-related physician claim relative to no DMT. Conversely, an elevated aHR was observed with the grouped second generation DMTs (aHR 1.47; 95% CI 1.16–1.85) although this association was only significant for natalizumab (aHR 1.59; 95% CI 1.19–2.11) when assessed separately. In comparison to first generation DMTs, the second generation drugs showed a 53% greater hazard for infection (aHR 1.53; CI 1.21–1.95). Infection-related hospitalisation was not significantly associated with any DMT group or class of drug compared to unexposed patients or when second generation DMTs were compared with first generation drugs. When assessing associations with individual infections, exposure to any DMT or any first generation DMT was associated with a lower hazard of pneumonia compared with no DMT exposure. Exposure to second generation DMTs was associated with a 58% and 68% increased hazard of an upper respiratory infection when compared to no exposure or first generation drug exposure, respectively. Natalizumab was the only second generation drug assessed separately and was associated with a higher hazard of an upper respiratory tract infection compared to no exposure (aHR 1.77; 95% CI 1.26–2.49).

*Comment* In this study, exposure to a second generation DMT was associated with an increase in the risk of infection, particularly with regard to natalizumab. First generation DMTs were not associated with an increased risk. Of note, IFN-β was associated with a lower risk of pneumonia, which the authors speculate could be due to its anti-viral effect. The reason for the increased risk of upper respiratory tract infections with second generation DMTs, and in particular natalizumab is unknown. It is acknowledged that the relatively small number of patients taking second generation DMTs may have limited the detection of differences.

Wijnands et al. J Neurol Neurosurg Psychiatry. 2018;89:1050–1056.

